# Supplementary material for: Surface Microbiome Profiling of Dental Elevators Using Third‐Generation Sequencing: Implications for Infection Control in Dental Practice
Source: Microbiologyopen. 2025 Dec 8;14(6):e70178. doi: 10.1002/mbo3.70178 (PMC12685768; doi:10.1002/mbo3.70178)
Supplement: Supplementary file 1 — customer_backup. [file MBO3-14-e70178-s001.zip › customer_backup/customer_backup/function_analysis/BugBase/treat/otu_contributions/family/treat.family.Stress_Tolerant.pdf]

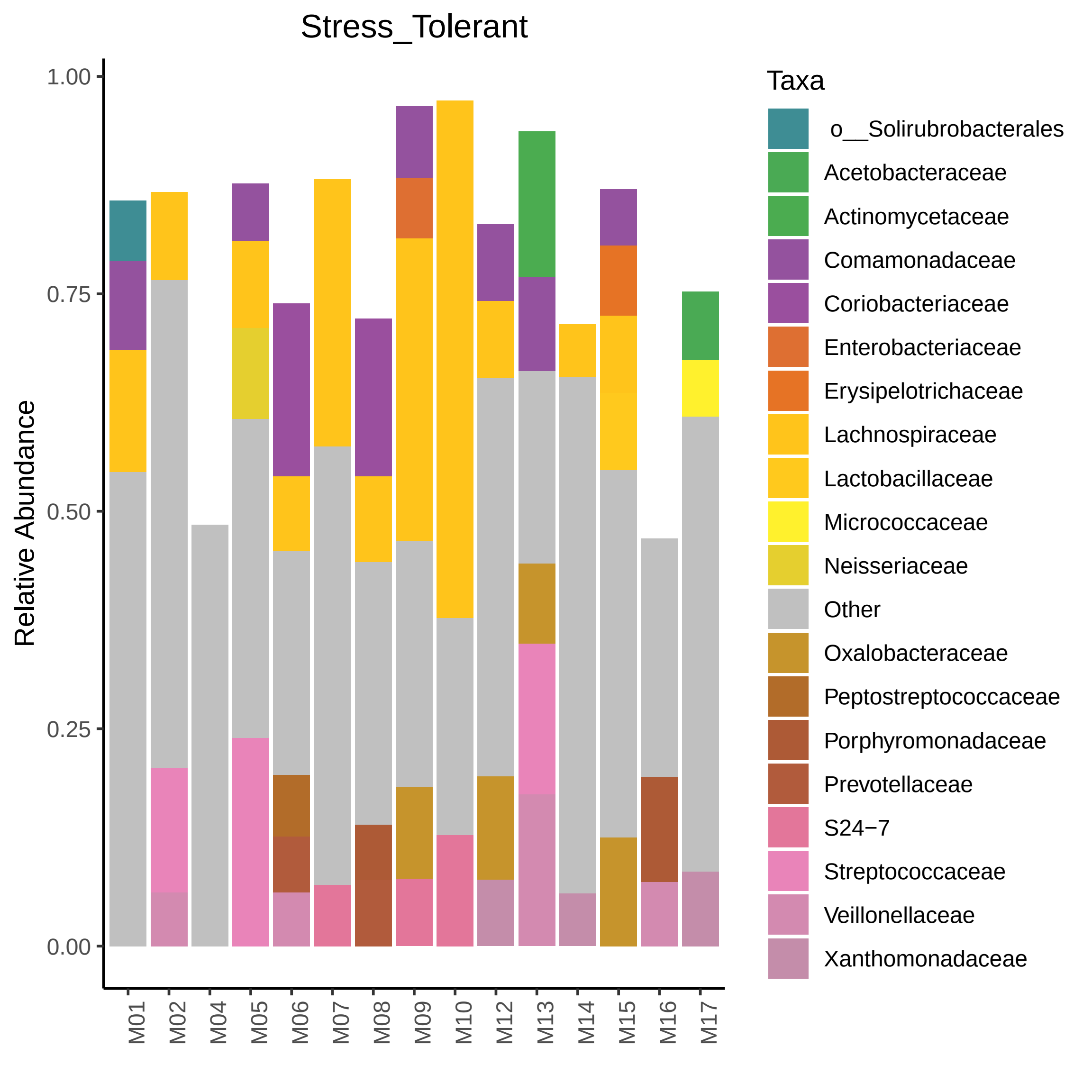
Stress_Tolerant
Relative Abundance
1.00
0.75
0.50
0.25
0.00
M01
M02
M04
M05
M06
M07
M08
M09
M10
M12
M13
M14
M15
M16
M17
Taxa
o__Solirubrobacterales
Acetobacteraceae
Actinomycetaceae
Comamonadaceae
Coriobacteriaceae
Enterobacteriaceae
Erysipelotrichaceae
Lachnospiraceae
Lactobacillaceae
Micrococcaceae
Neisseriaceae
Other
Oxalobacteraceae
Peptostreptococcaceae
Porphyromonadaceae
Prevotellaceae
S24−7
Streptococcaceae
Veillonellaceae
Xanthomonadaceae
